# Metal-Incorporated Mesoporous Silicates: Tunable Catalytic Properties and Applications

**DOI:** 10.3390/molecules23020263

**Published:** 2018-01-29

**Authors:** Anand Ramanathan, Bala Subramaniam

**Affiliations:** 1 Center for Environmentally Beneficial Catalysis, The University of Kansas, Lawrence, KS 66047, USA; anand@ku.edu; 2 Department of Chemical and Petroleum Engineering, The University of Kansas, Lawrence, KS 66045, USA

**Keywords:** Zirconium, tungsten, niobium, Lewis acid, mesoporous silica, dehydration, epoxidation, metathesis

## Abstract

A relatively new class of three-dimensional ordered mesoporous silicates, KIT-6, incorporated with Earth-abundant metals such as Zr, Nb, and W (termed as M-KIT-6), show remarkable tunability of acidity and metal dispersion depending on the metal content, type, and synthetic method. The metal-incorporation is carried out using one-pot synthesis procedures that are amenable to easy scale-up. By such tuning, M-KIT-6 catalysts are shown to provide remarkable activity and selectivity in industrially-significant reactions, such as alcohol dehydration, ethylene epoxidation, and metathesis of 2-butene and ethylene. We review how the catalytic properties of M-KIT-6 materials may be tailored depending on the application to optimize performance.

## 1. Introduction

### Need for Mesoporous Silica

It has been widely recognized that functionalized mesoporous silicates are necessary to process bulky substrates that are subject to diffusion limitations inside microporous catalytic materials, such as SAPOs and zeolites. In this regard, the successful synthesis of the MCM family of silicates by Mobil Corporation in 1992 [1,2] using micelles formed by a cationic surfactant as the templating agent, opened many possibilities and improved strategies (such as utilizing nonionic surfactant) [3] in preparing novel ordered mesoporous silicas with tunable pore structure and pore width. Recently, synthesis strategies for hierarchical porous materials [4] and mesoporous silica nanoparticles with tunable pores were comprehensively reviewed [5,6]. For catalytic applications, these mesoporous silicates need to be typically functionalized through incorporation of suitable active metal components.

Ordered mesoporous silicates, such as SBA-15 and KIT-6 [7,8], have relatively large pore width (compared to mesoporous silicates, such as FSM, HMS, and MCM) and are especially well suited to process bulkier feedstock molecules, such as those derived from biomass (e.g., fatty acid methyl esters and lignin) [9,10,11,12,13]. Further, the 3D pore architecture of KIT-6 materials tend to perform better than 2D pore architectures, such as SBA-15. For example, the more effective hydrotreating of gas oil over NiMo catalyst supported on KIT-6 compared to SBA-15 is attributed to the higher amount of MoS_2_ phases, along with better dispersion and higher concentration of the catalytically active edge sites (NiMoS) in the KIT-6 support [14]. Other examples include styrene epoxidation [15] and photocatalytic CO_2_ reduction to methane [16] over Ti-KIT-6 and Ti-impregnated KIT-6, respectively. Similarly, Pd-impregnated KIT-6 catalysts provide enhanced activity compared to either Pd/SiO_2_ or Pd/SBA-15 in the aerobic selective oxidation of crotyl and cinnamyl alcohols to their corresponding allylic aldehydes [17]. The markedly better performance of the M-KIT-6 catalysts is attributed to uniform dispersion of isolated metal species and the highly interconnected pore network, providing higher accessibility (~6400 pore entrances per μm^3^ for KIT-6 compared to ~290 pore entrances per μm^3^ for SBA-15). Further, propylsulfonic acid (PrSO_3_H)-functionalized KIT-6 materials are reported to be superior in the esterifications of propanoic, hexanoic, lauric, and palmitic acids with methanol, compared to either Amberlyst or SBA-15 catalysts [18]. A Science Citation Index^®^ analysis indicates that publications involving KIT-6 and SBA-15 have an average citation of 8.36 and 8.06, respectively, between 2013 and 2017 [19]. In this review, we focus mainly on the synthesis and catalytic applications of metal-incorporated KIT-6 including dehydration of lower alcohols, olefin metathesis, light olefin epoxidation, and lignin depolymerization.

## 2. Experimental

### 2.1. Scalable and Reproducible One-Pot Synthesis of M-KIT-6

Introduction of metals into mesoporous silica support (such as Al for providing acid sites and Ti for facilitating epoxidations) is carried out either by grafting, impregnation or vapor deposition of the metal onto the separately synthesized supports. Direct one-pot synthesis where the metal-incorporated support is formed in one step has also been reported. From a practical standpoint, one-step synthesis methods are preferred for their ease of preparation and better reproducibility compared to multi-step synthesis methods. Further, heteroatoms incorporated by one-pot synthesis techniques tend to be more uniformly distributed compared to grafting and impregnation techniques that result in a larger fraction of extra-framework sites at similar metal loadings.

Synthesis of KIT-6 is typically carried out under acidic conditions employing triblock copolymer Pluronic P123 (EO_20_-PO_70_-EO_20_) as template and n-butanol as co-solvent [8]. The pore size distribution can be tuned by varying the hydrothermal treatment temperature. In order to incorporate metal ions (such as Ti, Cu, Ga, La, B, Fe) under acidic conditions, the pH of the synthesis mixture is typically adjusted by lowering the acid concentrations (0.1–0.5 M HCl) [15,20,21,22,23]. We have successfully incorporated W, Zr, and Nb by adding the respective metal salts to the synthesis mixture of siliceous KIT-6 without any pH adjustments [24,25,26,27]. The molar synthesis gel composition is as follows: TEOS/M/P123/HCl/H_2_O/BuOH = 1.0/(0.01–0.10)/0.017/1.83/195/1.31 (where M=W, Zr and Nb). The organic templates were removed by calcination at 550 °C in a flow of dry air.

### 2.2. Unique Characteristics of M-KIT-6

**Figure 1:**
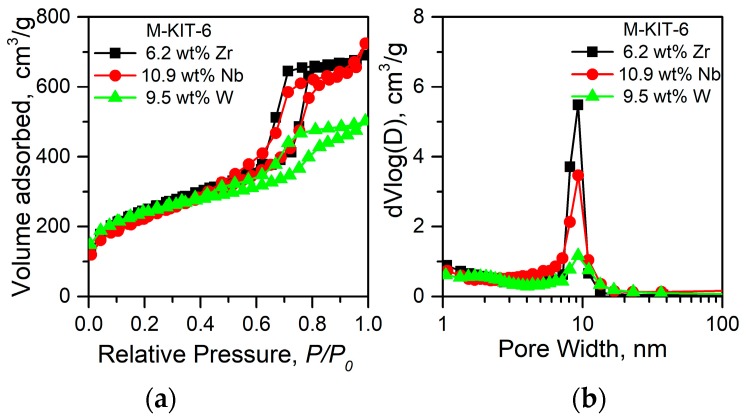
(**a**) Representative nitrogen sorption isotherms, and (**b**) pore size distributions of M-KIT-6, at various metal loadings.

**Figure 2:**
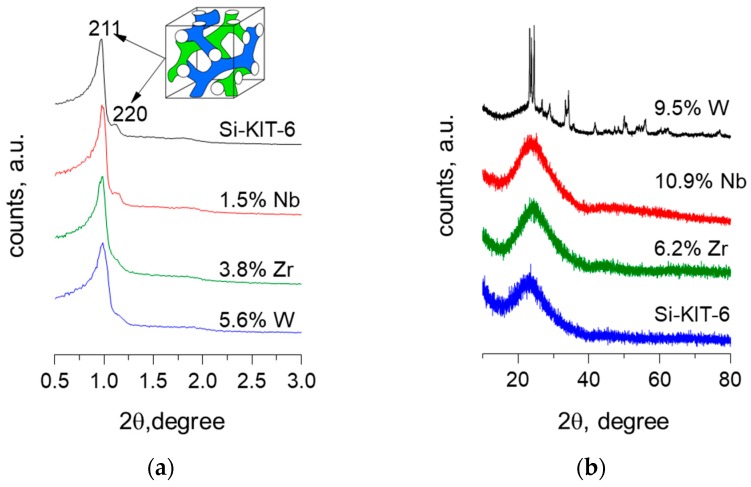
(**a**) Representative SAXS patterns, and (**b**) XRD diffractograms of M-KIT-6, at various metal loadings compared with pristine Si-KIT-6.

**Figure 3:**
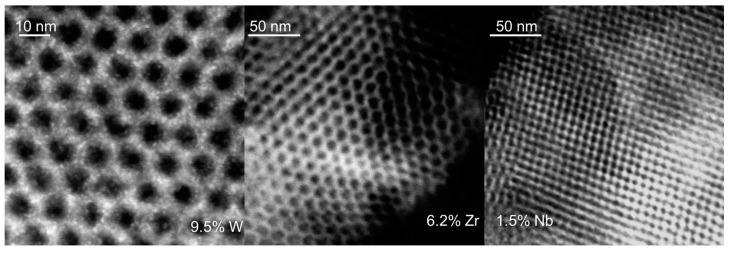
Representative STEM images of M-KIT-6 confirming ordered structure with highly-dispersed MOx species.

The surface area and pore volume of M-KIT-6 (M=W, Zr, Nb) materials range from 1013 to 536 m^2^/g depending on the metal loading (Table 1) [26,27,28]. Typical type IV isotherm with a narrow pore size distribution centered around approximately 9 nm was obtained irrespective of the metal content (Figure 1). At low metal loadings, an intense 211 reflection with a shoulder 220 reflection representative of cubic *Ia3d* type structure, along with long range mesoporous ordering was evidenced. At progressively higher metal contents, a decrease in the intensity of the 211 peak along with a lack of satellite reflections suggests loss of long range ordering of the mesopores (Figure 2a). These ordered pore structures were further confirmed by representative STEM images (Figure 3). Additionally, a homogeneous dispersion of W particles (as judged from the contrasting dots) is evident particularly in W-KIT-6. Further, from diffuse reflectance UV-VIS (not shown), highly dispersed isolated M^n+^ species are observed even at metal loadings up to 10 wt % along with oligomeric M_y_O_x_ species. In the case of Zr-KIT-6 and Nb-KIT-6 catalysts, neither bulk ZrO_2_ or bulk Nb_2_O_5_ species were evident in wide angle XRD spectra (Figure 2b) [26,27]. In contrast, extraframework WO_3_ species were evident in W-KIT-6 materials even at low W loadings (3–5 wt %) [24,28]. This may be because the tungsten ion is too large to be easily inserted into the silicate framework. 

**Figure 4:**
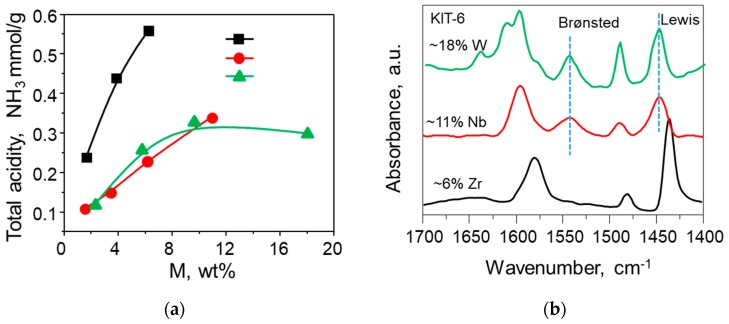
(**a**) Comparison of total acidity variation with metal loading in KIT-6 catalysts; and (**b**) FTIR spectra of adsorbed pyridine over M-KIT-6 at relatively high metal loadings.

In general, an increase in metal content results in an increase in the total number of acid sites. For instance, the total acidity of Zr-KIT-6 and Nb-KIT-6 materials increases linearly with an increase in metal loading (from 1 to 10 wt %), with the increase being more pronounced in Zr-KIT-6 (Figure 4a). In contrast, the normalized acidity of W-KIT-6 materials reaches a maximum value of approximately 10 wt % due to the formation of bulk crystalline WO_3_ species at higher loadings that have negligible acidity.

Figure 4b compares the Lewis and Brønsted acidities of the M-KIT-6 materials. The Zr-KIT-6 materials are predominantly Lewis acidic at all metal loadings up to 6 wt % [27]. Nb-KIT-6 materials exhibit Lewis acidity of much lower strength compared to Zr-KIT-6 [26,29]. However, a pronounced increase in Brønsted acid sites is observed at higher Nb loadings. Even though the total acidity of W-KIT-6 materials is similar to Nb-KIT-6 materials, the number of Brønsted acid sites is higher compared to Nb-KIT-6 (Figure 4b). In the following sections, we discuss how the tunable acidities of metal-incorporated KIT-6 materials may be exploited in a variety of industrially relevant chemistries.

## 3. Results and Discussion

### 3.1. Applications of M-KIT-6 Materials in Gas Phase Reactions

#### 3.1.1. Selective Dehydration

The dehydration of bioethanol and mixed bioalcohols to make renewable olefins has been receiving increased interest in recent years [30,31]. Zr-KIT-6 materials are predominantly Lewis acidic and have been shown to be effective for dehydration of lower alcohols, such as isopropanol and ethanol [27,32]. Almost total conversion was achieved for isopropanol with nearly total selectivity to propene. In comparison, propene selectivity ranges from 21% to 76% over Lewis acidic γ-Al_2_O_3_ or ZrO_2_ catalyst [33]. At similar operating conditions, only 15–30% ethanol conversion was achieved over Zr-KIT-6 in the 300–360 °C range, similar to that observed with ZrO_2_. However, much higher ethylene selectivity (60–80%) was achieved with Zr-KIT-6 compared to ~40% with commercial ZrO_2_. Further, the intrinsic rate constants for both isopropanol and ethanol dehydration on Zr-KIT-6 catalysts (with different Zr loadings) overlap when normalized with the acid sites in the catalyst samples. The corresponding activation energies for isopropanol and ethanol dehydration to form propylene and ethylene, respectively, were found to be 49 ± 1 kJ/mol and 79 ± 1 kJ/mol. Despite possessing more Brønsted acid sites than Zr-KIT-6, Nb-KIT-6 materials showed lower selectivity towards ethylene [29].

**Figure 5:**
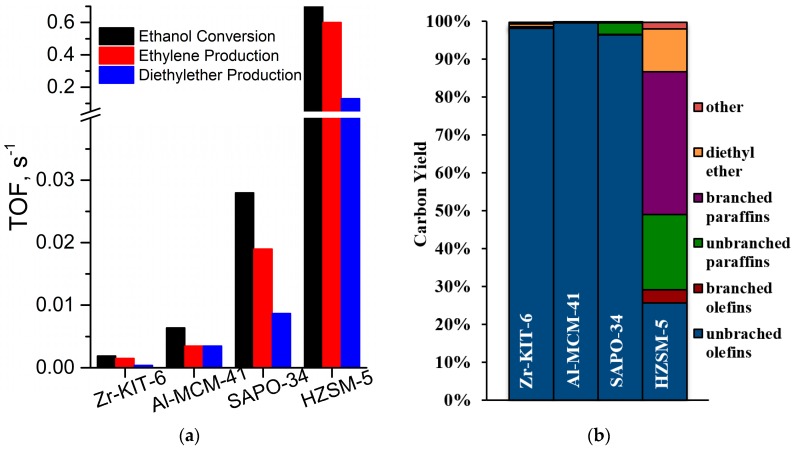
(**a**) TOFs for ethanol conversion and dehydration product yields at 300 °C; and (**b**) carbon yield from mixed alcohol dehydration experiments with the ethanol-rich mixed alcohol feed at 350 °C. ‘Other’ products are carbon monoxide, carbon dioxide, propanal, and methanol. Data represent the average of replicate data points at WHSV = 0.32 h^−1^ [31].

As shown in Figure 5a, catalysts possessing a higher fraction of strong Brønsted acid sites, such as H-ZSM-5 and SAPO-34, displayed much greater ethanol dehydration activity at similar operating conditions compared to mesoporous materials, such as Al-MCM-41 and Zr-KIT-6 [31]. However, as shown in Figure 5b, H-ZSM-5 yielded several side products (branched and unbranched paraffins, branched olefins, aromatics, etc.) in significant quantities, in addition to ethylene and diethyl ether, while zeolites H-MOR [34] and H-BEA [35], with similar strong Brønsted acid sites are more selective for ethylene [36]. The enhanced formation of ethylene and diethyl ether in zeolites is generally attributed to the presence of very strong Brønsted acid sites, as well as the confinement effect [36]. The generation of stronger acid sites by metal incorporation benefits ethylene selectivity by not only increasing the reaction rates over the acid sites, but also poisoning the basic sites that are responsible for the formation of dehydrogenation products [37].

These results indicate that the relative populations of Brønsted acid sites and Lewis acid sites, as well as the strength of the acid sites are tunable by varying the content and type of incorporated metal. Such tunability in turn, dictates product selectivity. The use of metal-exchanged mesoporous catalysts with tunable acidity for the dehydration of bulky biomass-derived sugar alcohols (such as glycerol, sorbitol, glucose) to valuable products (such as acrolein, anhydrosorbitol, isosorbide, 5-hydroxymethylfurfural (HMF), levulinic acid, or gamma-valerolactone, etc.) is attractive [38,39], but not fully explored as yet.

#### 3.1.2. Olefin Metathesis 

Shale gas extraction produces collateral amounts of ethane and other lower alkanes (approximately 16 wt %). The dominant presence of ethane is expected to result in a copious supply of ethylene. It is anticipated that the metathesis reaction involving ethylene and butenes (the dimerization product of ethylene) will play a significant role in partly meeting the increased demand for propylene. For the heterogeneous metathesis reaction, the catalysts most frequently reported in the literature contain W, Mo, or Re as active species supported on SiO_2_ or Al_2_O_3_ supports [40]. Current industrial technologies employ WO_3_/SiO_2_ catalyst for this metathesis reaction at *T* > 260 °C and 30–35 bar [41]. We have shown that W-incorporated mesoporous catalysts (W-KIT-6, W-KIT-5, and W-SBA-16) resulted in superior propylene yield compared to WO_3_-impregnated catalysts (WO_3_/SiO_2_ and WO_3_/KIT-6) for ethylene and 2-butene metathesis [28] due to a higher dispersion of active tungsten precursors in the direct method of W-incorporation. A stable propylene yield at 450 °C was observed over these catalysts in a fixed-bed reactor for a duration of 7 h.

**Figure 6:**
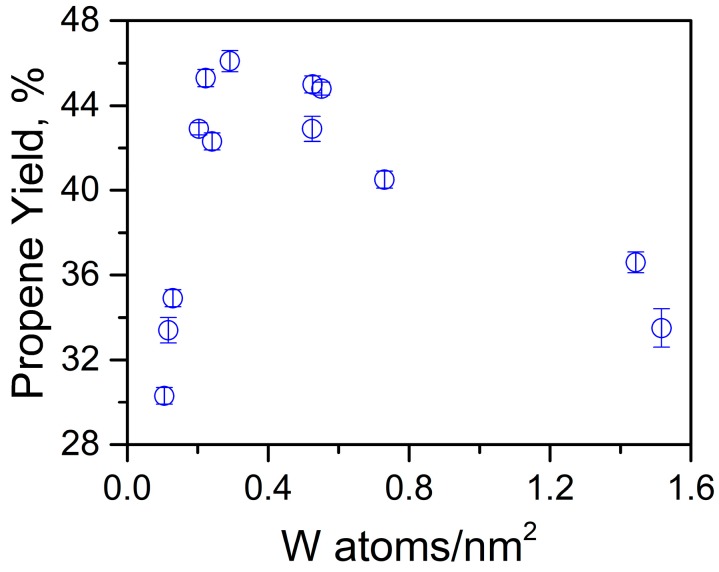
Comparison of propene yield with normalized W loading in various cubic mesoporous silicate supports (KIT-6, KIT-5, and SBA-16).

Interestingly similar propene yield (~45%) at an optimum W loading (6–10 wt %) and catalyst acidity (0.2–0.3 mmol NH_3_/g) was noticed irrespective of the support (KIT-6, KIT-5, or SBA-15), suggesting that the structural differences among these catalysts do not play a major role in their performance. Normalizing these results with the number of W atoms/nm^2^ in these supports (KIT-6, KIT-5 or SBA-15), revealed that maximum propylene yield of about 44 ± 2% was achieved between the ranges of 0.2 and 0.5 W atoms/nm^2^ (Figure 6).

Delaying the W addition by 2 h (as opposed to adding the W source simultaneously with the Si source) during one-pot synthesis showed an increase in propene yield (~59%) compared to W-KIT-6 prepared by conventional method. XPS results reveal that the delayed W addition produces a greater fraction of active and accessible active W species on the KIT-6 surface (Table 2) [28]. Similar enhancements in propene yield (~55%) was obtained over WO_3_/SiO_2_ catalyst (~11.7% W) prepared by an aerosol-assisted sol–gel process [42]. Ternary Si-Al-W mixed oxide catalysts prepared by a one-step aerosol process showed stable metathesis activity (38% propene yield) at much lower temperature (250 °C) compared to tungsten impregnated on commercial silica–alumina or aerosol-made silica–alumina powder [43]. These results clearly show that catalyst synthesis procedures have a significant effect on catalyst performance.

In yet another example, bimetallic W-Al-KIT-6 catalysts are shown to be stable and active catalysts for the 1-butene+ethylene metathesis to propene [44]. Without a 1-butene isomerization catalyst (MgO/Al_2_O_3_) preceding the metathesis catalyst, a higher temperature of 450 °C was necessary for the bimetallic W-Al-KIT-6 catalyst to achieve 78.2% 1-butene conversion and 82.4% propene selectivity [44]. Optimum W and Al contents (5.06 and 0.88 wt % respectively) were necessary to maximize W dispersion of W species and acidity, resulting in improved propylene yields [44].

Tungsten forms two types of isolated sites on silica support: mono-oxo (O=WO_4_) and dioxo (O=)_2_WO_2_, both representative of W^6+^ species with the latter being the dominant and most likely precursor under reaction conditions [45,46]. Catalysts pretreated in the presence of either He or propene slowly transform the mono-oxo sites to dioxo sites, thereby reducing the activation period usually observed in these catalysts [45]. Interestingly WO_3_/SiO_2_ catalyst ion-exchanged with NaOH also displayed a shorter induction period with a marginal decrease in the isomerization activity [47]. While the metathesis mechanism under reaction conditions is not yet fully understood, it is clear that the metathesis activity and selectivity are tunable with acidity in metal-incorporated silicates and remains to be explored in more detail, especially with regard to bimetallic formulations.

### 3.2. Applications of M-KIT-6 Materials in Liquid Phase Reactions

#### 3.2.1. Liquid Phase Epoxidations

Creating exclusive Lewis sites in microporous and mesoporous silicates is highly desirable, especially for olefin epoxidations, as even a small number of Brønsted acid sites could lead to ring opening of the epoxide, as well as decomposition of the oxidant (H_2_O_2_). Recently, metal precursors were reacted with delaminated zeolites to create isolated Lewis acid sites [48]. Ti incorporated in this manner was shown to exhibit higher activity compared to Ti/SiO_2_ for epoxidation of cyclohexene and 1-octene with TBHP. Nb/SiO_2_ catalyst was found to be superior compared to Ti/SiO_2_ catalyst for limonene epoxidation with H_2_O_2_. The catalysts were prepared by dry-impregnating organometallic precursors under solventless conditions into commercial Davisil silica. In fact, Ti/SiO_2_ was effective only in the presence of TBHP [49]. Further, niobium silicates prepared by impregnation techniques showed better stability compared to those prepared by co-precipitation techniques [50]. More recently, the selective formation of either isolated Nb species or oligomeric Nb species in the mesoporous silica matrix was achieved with either ammonium niobate(V) oxalate hydrate or niobium(V) ethoxide in combination with acetylacetone (acac), respectively [51]. For carene as a substrate, these catalysts demonstrate nearly total epoxidation activity with H_2_O_2_, but only 26% epoxide selectivity with TBHP [51]. Grafted Nb/SiO_2_ catalysts [52] were demonstrated for the epoxidation of a variety of unsaturated cyclic and terpenic compounds, such as cyclohexene, 1-methylcyclohexene, limonene, carveol, terpineol, isopulegol, carvotanacetol, carvone, as well as squalene and isopulegyl acetate in the presence of H_2_O_2_. These catalysts displayed high epoxide yields (up to 73%) and excellent chemo-selectivities to the desired epoxides (up to 98%) in relatively short reaction times (1 h). In addition, rather unexpectedly, epoxidation of the less electron-rich, exocyclic C=C double bond in limonene and carveol was also observed. The grafted Nb/SiO_2_ catalysts showed a gradual decrease in activity during recycle runs likely because of surface poisoning, pore plugging, and site blocking by organic byproducts. It must be noted that many of the reported studies do not report the extent of H_2_O_2_ decomposition in the presence of Brønsted acid sites. 

A major grand challenge in industrial chemistry continues to be the effective utilization of ethylene during its epoxidation to ethylene oxide (EO) wherein 10–15% of the ethylene is burnt as CO_2_ in the current technology [53]. Remarkable ethylene epoxidation activity was observed on Nb-KIT-6 and W-KIT-6 catalysts at mild temperatures where CO_2_ formation as byproduct is avoided [54]. The reaction was carried out at 35 °C and 50 bar in an ethylene-expanded liquid phase with H_2_O_2_ as an oxidant and methanol as the solvent. The epoxidation mechanism usually involves metal-peroxo complex formation with H_2_O_2_ that is then transferred to the olefin for epoxide formation. Raman spectra reveal that both niobium- and tungsten-based silicates form such complexes in the presence of H_2_O_2_ [55,56]. The EO yield over Nb-KIT-6 (234–794 g EO/h/kg Nb) is significantly higher than those observed with W-KIT-6 materials (34–152 g EO/h/kg W) (see Table 3). In general, the EO yield decreased with an increase in metal loading. These results imply that isolated M^n+^ species are active for EO formation, whereas extra framework species observed at higher metal loading are not as active. The selectivity for EO is in the range of ~80% over W-KIT-6 and 46–73% with Nb-KIT-6. The observed EO yields are comparable with those reported for homogeneous Re-based (1610–4970 gEO/h/kg Re) [57] and conventional heterogeneous Ag-based catalysts (700–4400 gEO/h/kg Ag) [58,59]. However, H_2_O_2_ decomposition (~80%) and metal leaching caused by Brønsted acid sites in these catalysts are major drawbacks [54]. Clearly, the challenge is to synthesize M-KIT-6 materials that are exclusively Lewis acidic by passivating the Brønsted acid sites. To address the stability of KIT-6, and to improve the utilization of H_2_O_2_, we studied Nb-TUD-1 as a catalyst and found that it showed superior activity (~2500 g EO/h/kg Nb) compared to other Nb- or W-based silicates [59]. The EO productivity was further improved to ~4000 g EO/h/kg Nb by decreasing the Brønsted acid sites achieved by lowering the Nb loading. Nevertheless, about 60% of Nb was leached after initial run [59]. To further thwart the Brønsted acidity, Nb-TUD-1 was capped with ion pair agents or covalent bonding groups [60]. Among these groups, covalently-bound capping agents showed improved stability. Specifically, the benzylated form of Nb-TUD-1 catalyst displayed improved H_2_O_2_ utilization toward EO formation (~60–71%) and significantly reduced Nb leaching (~3%), while maintaining structural integrity and providing high EO selectivity (>98%). Thus, Nb-based mesoporous catalysts show much promise for selective, ethylene epoxidation in a liquid phase using H_2_O_2_ as oxidant, similar to industrial propylene oxide technologies.

#### 3.2.2. Liquid Phase Lignin Depolymerization 

Currently, the paper and pulp industry generates approximately 50 million tons of Kraft lignin annually [61]. In 2014, 33 million gallons of cellulosic ethanol was produced commercially in the United States, meeting the volume mandated by the Renewable Fuel Standard for the first time [61,62]. In 2016, this production increased to 230 million gallons [62]. Significantly, for every liter of cellulosic ethanol produced, approximately 0.5–1.5 kg of lignin will be co-generated depending on the nature of lignocellulose used in the process [61]. The controlled deconstruction of lignin into aromatic platform chemicals is essential for ensuring the economic viability of next generation of cellulosic ethanol bio-refineries. However, lignin valorization remains a challenge due to its inherent heterogeneity, recalcitrance, and complex three-dimensional polymeric structure composed of C-C and C-O bonds. Most reported studies employ model lignin compounds, such as phenolic monomers and dimers. While these studies are useful in promoting a fundamental understanding of bond cleavage mechanisms with specific catalysts, they are not directly applicable to the deconstruction of real lignin [63]. Direct production of aromatics from lignin without the use of externally-added hydrogen is a desirable strategy, considering the high cost and large quantities of hydrogen required in some of these processes. In this regard, Zr-KIT-6 and Zr-KIT-5 catalysts that are predominantly solid Lewis acids show superior performance for lignin depolymerization [13] compared to published reports with other acidic catalysts, yielding phenolic monomers that are promising precursors for upgrading to aromatic chemicals and fuels. In particular, Zr-KIT-5 was found to have superior activity (97% lignin conv. and 65% yield of THF soluble products) compared to H-ZSM-5 (92% lignin conv. and 54% yield of THF soluble products) [64]. Further, the yield of solid product residue (31%) over H-ZSM-5 was greater than that observed with Zr-KIT-5 (24%). This may be attributed to the fact that the micropores of H-ZSM-5 may hinder effective deconstruction of the bulky lignin to monomers. In contrast, the three-dimensional mesoporous nature of Zr-KIT-5 facilitates effective breakdown of bulky molecules leading to the higher yield of monomers. It is also possible that the predominantly Brønsted acidic H-ZSM-5 may favor repolymerization of the formed monomers leading to high yield of condensate. On other hand, the dominant Lewis acidity of Zr-KIT-5 may minimize repolymerization. Nevertheless, structural degradation and acidity loss were evident in the spent Zr-KIT-5 catalysts primarily due to a lack of hydrothermal stability and the presence of residual inorganic impurities (Na_2_SO_4_) in the dealkaline lignin.

## 4. Catalyst Deactivation

Catalyst deactivation occurs mainly by coke formation during dehydration of simpler alcohols and olefin metathesis on M-KIT-6 catalysts. This is attributed to the presence of Brønsted acid sites. For example, H-ZSM-5 possessing strong Brønsted acid sites yields side products (branched alkanes and alkenes) due to cracking/isomerization/condensation reactions of hydrocarbon reactants, intermediates, and/or products. The heavier products tend to accumulate in the pores of the catalyst causing catalyst deactivation. In contrast, stable activity was observed for ethanol or isopropanol dehydration over Zr-KIT-6 that possesses predominantly Lewis acid sites [27,32]. During the metathesis of 2-butene and ethylene, a moderate decrease in the 2-butene conversion from 84.2% to 79.4%) was observed over W-KIT-6 during 120 h. Correspondingly, the propene yield decreased from 79.2 to 72.6% [28]. Solid-state ^13^C NMR analysis of the spent catalyst revealed the presence of oligomers [poly(1-butene)] due to the polymerization of 1-butene formed from the isomerization of 2-butene). The catalyst, however, regained its activity following regeneration in air at 550 °C [65].

In the liquid phase epoxidation involving H_2_O_2_, it is widely accepted that the tetrahedrally-coordinated metal (Lewis site) in the silica matrix forms a metal peroxo complex by reaction with H_2_O_2_ which then interacts with olefinic substrates to form the corresponding epoxide [54]. Computational chemistry studies of the interaction of H_2_O_2_ on a simple niobyl silicate site (Nb=O, representative of NbO_4_ units) and niobium hydroxide (Nb-OH, representative of Brønsted acid sites) silicate indicate that the presence of Brønsted acid sites causes H_2_O_2_ decomposition with concomitant Nb leaching [59]. Indeed, the design of catalysts with reduced Brønsted acidity increases H_2_O_2_ utilization towards epoxide formation while simultaneously reducing metal leaching [60,66].

## 5. Summary and Outlook

Metal-incorporated mesoporous silicates, composed of inexpensive Earth-abundant metals, show much potential for development and practical implementation. Their applications in converting feedstocks derived from biomass and natural gas liquids (NGLs) have the potential to yield novel catalytic processes. However, much fundamental work remains to be done. For example, characterization of the surface oxides (by X-ray photoelectron spectroscopy and other techniques) is critical to a fundamental understanding of the correlation between oxide species and the distribution of Lewis and Brønsted acid sites. Such an understanding is essential to the rational design of catalysts to achieve the desired activity, selectivity and stability for the various potential applications. Further, the development of hydrothermally stable catalysts is particularly essential for liquid-phase reactions in aqueous media.

Optimized formulations of metal-incorporated mesoporous silicates (i.e., with optimal pore sizes and tuned acidities) are particularly well suited for catalytic processing of bulky biomass substrates in applications such as glycerol dehydration, epoxidation of long-chain fatty acid methyl esters, and lignin depolymerization. Complementary computational studies are also needed to better understand the origins of acidity upon metal incorporation and specific catalytic chemistry to guide optimal catalyst design. Lastly, the single-pot nature of the synthesis method enhances the potential for successful scale-up of these catalysts and, therefore, their practical viability.

## Figures and Tables

**Table 1:** Physicochemical properties of M-KIT-6 catalysts.

KIT-6 (Si/M) ^a^	Si/M ^b^	M wt % ^b^	S_BET_^c^ m²/g	V_tp_^d^ cm^3^/g	d_P_, _BJH_^e^ nm	Total Acidity ^g^ mmol NH_3_/g
Si-KIT-6	-	-	1013	1.38	9.3	0.04
Zr-KIT-6(100)	92	1.6	980	1.65	9.3	0.19
Zr-KIT-6(40)	39	3.8	881	1.42	9.3	0.40
Zr-KIT-6(20)	23	6.2	810	1.07	9.3	0.49
Nb-KIT-6(100)	98	1.5	997	1.46	9.3	0.11
Nb-KIT-6(40)	41	3.4	991	1.29	9.3	01.5
Nb-KIT-6(20)	21	6.1	926	1.28	9.3	0.23
Nb-KIT-6(10)	9.8	10.9	804	1.12	9.3	0.34
W-KIT-6(100)	104	2.9	880	1.03	9.3	0.13
W-KIT-6(40)	51	5.6	764	0.81	9.3	0.26
W-KIT-6(20)	29	9.5	661	0.69	9.3	0.33
W-KIT-6(10)	14	18.0	536	0.60	8.1	0.33

^a^ Molar ratio in the synthesis gel, ^b^ Actual molar ratio and M wt % in sample determined by ICP-OES, ^c^ S_BET_ = BET Specific Surface Area from adsorption isotherm at *P*/*P*_0_ between 0.05 and 0.30, ^d^ Vt_P_ = Total pore volume at 0.99 *P*/*P*_0_, ^e^ d_P,BJH_ = pore width calculated from N_2_ adsorption isotherms using the BJH model, ^g^ From ammonia TPD measurements.

**Table 2:** Atomic ratio of W/Si in W-KIT-6 catalysts measured from XPS and ICP revealing surface enrichment [28].

Catalyst	W-KIT-6 (8.7)	W-KIT-6 (2 h, 9.2)
W (at %) ^1^	2.74	3.86
Si (at %) ^2^	97.3	96.1
W/Si Atomic Ratio ^3^	0.0282	0.0402
W/Si Atomic Ratio from ICP	0.03204	0.03421
W/Si increment from XPS, %	-	42.6
W/Si increment from ICP, %	-	6.8
Apparent TOF (mmol_propene_ mol w^−1^ s^−1^)	3.58	4.69
Propylene yield, %	42.9	59.2

^1^ Calculated from the peak area of W_4f_, ^2^ Calculated from the peak area of Si_2p_, ^3^ Atomic ratio.

**Table 3:** Epoxidation activity of ethylene over M-KIT-6 and Nb-TUD-1 catalysts (reaction conditions: MeOH = 624 mmol, H_2_O_2_ = 118 mmol, AN = 4.9 mmol, catalyst loading = 500 mg, T = 35 °C, Ethylene P = 50 bar (maintained constant), t = 5 h, 1400 rpm).

Catalyst	M wt%	P_EO_^a^ (±3%)	*S*_EO_ % ^b^ (±3%)	*X*_H_2_O_2__ % ^c^ (±3%)	*U*_H_2_O_2__ % ^d^ (±3%)	*Leaching* (±5%)
W-KIT-6	17.9	34.4	81.4	10.2	3.6	74.1–100
	9.4	43.4	80.0	6.4	3.9	
	5.7	66.5	84.0	6.0	3.5	
	2.2	152.6	80.0	4.2	5.0	
Nb-KIT-6	13.4	234	46.8	17.1	18.8	33.7
	7.2	340	52.7	17.1	13.1	32.4
	3.7	513	62.6	17.5	8.4	61.6
	1.5	794	73.4	11.2	7.1	72.4
Nb-TUD-1	4.0	1186	89.1	15.7	8.6	52.5
	1.4	2539	87.8	12.8	9.1	60.8
	0.88	4304	91.7	5.8	20.6	62.2
Benzylated Nb-TUD-1		597	98.7	0.65	59.7	3.1

^a^ Productivity and ^b^ selectivity of ethylene oxide; ^c^ Conversion and ^d^ utilization of H_2_O_2_.
