# Erratum: Ramanathan, A.; et al. Metal-Incorporated Mesoporous Silicates: Tunable Catalytic Properties and Applications. *Molecules* 2018, *23*, 263

**DOI:** 10.3390/molecules23040853

**Published:** 2018-04-09

**Authors:** Anand Ramanathan, Bala Subramaniam

**Affiliations:** 1 Center for Environmentally Beneficial Catalysis, The University of Kansas, Lawrence, KS 66047, USA; anand@ku.edu; 2 Department of Chemical and Petroleum Engineering, The University of Kansas, Lawrence, KS 66045, USA

The authors wish to make the following change to their paper [1]. The Acknowledgments section is incorrect in the published paper. The correct Acknowledgments should be as follows:

The authors gratefully acknowledge partial support for this work from the following grants: the joint National Science Foundation and Environmental Protection Agency program Networks for Sustainable Material Synthesis and Design (NSF-EPA 1339661) and the National Science Foundation (OIA-1539105).

We apologize for any inconvenience caused to the readers by this mistake.
